# Impact of Ethnicity on COVID-19 Related Hospitalizations in Children During the First Pandemic Wave in Northern Italy

**DOI:** 10.3389/fped.2021.625398

**Published:** 2021-02-04

**Authors:** Roberto Baronio, Lucia Savaré, Jessica Ruggiero, Beatrice Crotti, Angelo Mazza, Gian Luigi Marseglia, Icilio Dodi, Claudio Cavalli, Richard Fabian Schumacher

**Affiliations:** ^1^Department of Clinical and Experimental Sciences, University of Brescia, Brescia, Italy; ^2^Papa Giovanni XXIII Hospital, Bergamo, Italy; ^3^Fondazione Ospedale San Matteo, Pavia, Italy; ^4^Pediatria Generale e d'Urgenza, Ospedale dei Bambini Pietro Barilla, Parma, Italy; ^5^Pediatrics, Ospedale Maggiore, ASST Cremona, Crema, Italy; ^6^Children's Hospital, ASST Spedali Civili, Brescia, Italy

**Keywords:** SARS-CoV-2, children, population, ethnicity, COVID-19, hospitalization

## Abstract

There is increasing evidence that black people and other minorities have a higher incidence of severe COVID-19 disease, but little is known about the situation of children, especially in Europe. In general children are less infected and if so, frequently show mild or asymptomatic disease, making conclusions difficult. We collected data on SARS-CoV-2 associated hospitalizations in a well-defined population of 550,180 children up to 15 years in five hub-centers during the “first wave” at the heart of the pandemic in Northern Italy. Among the 451,053 Italian citizens 80 were hospitalized as compared to 31 out of 99,127 foreign citizens, giving a significantly higher risk (odds ratio 1.76; 95% CI: 1.16–2.66) for the foreign children. The risk was highest for children of African ethnicity as compared to Italians with an odds ratio of 2.76 (95% CI: 1.56–4.87). None of the patients deceased. There was no significant difference in age (thou infants regardless of ethnicity had a 10-fold higher risk), sex, length of hospitalization or comorbidities, namely overweight. As bureaucratic, cultural and information barriers mostly affect preventive and adult services and considering that in contrast to other countries, in Italy pediatric care is guaranteed free of (out-of-pocket) charge to all people <16 years, and hospitals are densely spaced, access to health care seems to be a minor problem. Thus, other possible root causes are discussed. We believe that this is an unbiased starting point to understand and overcome the reasons for the higher risk those children experience.

## Introduction

Since the outbreak of the COVID-19 pandemic, more than 44 million cases and 1 million fatalities have been reported worldwide up to the end of October 2020 (1).

The dynamic of spread and the severity of COVID-19 remarkably vary among different geographical regions: the Americas account for almost 50% of cases and more than 50% of deaths, followed by Europe (23% of cases, 24% of deaths) and South-East Asia (20% of cases, 12% of deaths), while Africa has seen only 1 million of cases (3%) and 29,491 deaths (2.5%) (1).

Interestingly, an opposite trend is reported within the most affected countries (namely the USA), where people of non-white ethnicity – and especially those of African American ethnicity - have a much higher incidence and mortality from COVID-19 than that observed in the population of Caucasian ethnicity. The explanation is probably related to the socio-economic differences aggravated by a health system that does not offer equal care and services to all members of the society (2). This theory is supported by a recent analysis conducted in Brazil that reported an increased mortality in regions with lower levels of socioeconomic development and especially among black Brazilians and those of mixed ethnicity (3). But not only in the Americas, also in Europe ethnic minorities seem to be disproportionally hit as a recent report from the Public Health England showed that COVID-19 mortality was greater in Black, Asian and Minority Ethnic (BAME) people than in white British people (4). An English research highlighted that, in UK, the two thirds of the 106 healthcare workers who died of COVID-19 until April 2020 were from ethnic minorities (5). However, studies aimed to investigate the relationship between ethnicity, comorbidities, socioeconomic position and greater severity of COVID-19 did not find a clear association (6). Moreover, some reports highlight a possible role of biological factors such as Vitamin D deficiency (7), ACE1 gene I/D polymorphisms and higher thrombotic risk in Black people (8), even if this is in contrast with the lower disease burden in sub-Saharan Africa (9). Therefore, whether this heterogeneity in COVID-19 burden is entirely due to genetic susceptibility, socioeconomic or environmental factors is not completely clear yet.

Pediatric data are scarce, though there is some evidence showing again a higher rate of morbidity and severity of COVID-19 in African American children (10, 11), in Afro-Caribbean (12), and in BAME communities (13).

Italy has been the first European country to be massively affected by the COVID-19 pandemic, counting 547,532 cases and with more than 37,000 deaths one of the highest case fatality rates. Pediatric cases (<19 years old) increased over time and account now for 8.8% of the total, but only 4 deaths occurred in that age range (14).

To our knowledge, there are no published data regarding the ethnicity of SARS-CoV-2 infected children living in Italy. In contrast to most other countries, Italy's National Health Service guarantees every child - regardless of nationality, citizenship or ethnic origin - a pediatrician, and whenever necessary, pediatric out- and in-patient care in one of the densely spaced hospitals completely free of charge (at the point of service) and equal for all (15, 16). For acute care just access the emergency room, no need to enroll with a pediatrician before. Thus, some of the logistic, bureaucratic and economic confounders encountered elsewhere, can be excluded. Cultural beliefs and shame, both well-known barriers especially in the context of adult and chronic diseases, are of less concern in our acute, pediatric context. Furthermore, as children in general are healthier in terms of the typical adult comorbidities (diabetes, obesity, heart disease, hypertension, smoking etc.) that impact on COVID-19, analyzing this particular patient group may reveal important information.

With this paper, we thus wanted to show if and how ethnicity may affect COVID-19 incidence and severity in children in Italy, especially those of African ethnicity. The aim of our multicenter retrospective study is to report epidemiological, demographic, and clinical data on children admitted to pediatric hospitals with SARS-CoV-2 infection from February 24 to July 10, 2020, paying particular attention on the “first wave” of the viral spread.

We decided to focus on five Hospitals that found themselves at the heart of the pandemic in Northern Italy, and which represented each the only designated local Hub Center, where all pediatric COVID-19 cases of a specific district, namely Bergamo, Brescia, Cremona, Parma, and Pavia were hospitalized.

## Methods

We included all pediatric patients (aged 0–15 years and 364 days – as older children might be seen and hospitalized also in adult wards) testing positive for SARS-CoV-2 real time-polymerase chain reaction (RT-PCR), who required hospital care between February 24 and July 10 at the Pediatric Units of Brescia (Ospedale dei Bambini - Spedali Civili), Cremona (Ospedale Maggiore), Bergamo (Ospedale Papa Giovanni XXIII), Pavia (Fondazione Ospedale San Matteo), and Parma (Ospedale dei Bambini Pietro Barilla). We choose those hospitals as they were all the one single pediatric COVID-19 Hub Centers in their respective Provinces, thus including all pediatric COVID-19 patients <16 years in a precisely-defined population of >500,000 children – among them roughly 100,000 of foreign ethnicity - at the heart of the first wave of the pandemic in Italy. Precise data for yearly age cohorts of Italian and foreign children are published as of December 31st, 2019 by the Italian National Institute of Statistics (ISTAT) and were retrieved for each Province at www.tuttitalia.it (16). The relative percentages published there of the foreign citizens according to different geographic areas (Europe, Asia, Africa, America, and Oceania plus statelessness) in the different provinces were applied to the pediatric cohorts of each Province and used as denominator.

According to shared hospital guidelines, RT-PCR for SARS-CoV-2 on rhino-pharyngeal swab was always performed in the presence of fever, respiratory or gastro-intestinal symptoms, as well as in case of a history of close contacts with COVID-19 patients. From April, rhino-pharyngeal samples were systematically collected as screening test at admission to the hospital, for epidemiological purposes and containment of the pandemic (see Table 1). According to regional directives, the RT-PCR for SARS-CoV-2 on swabs performed in the first wave of the pandemic analyzed three genes: RdRP (RNA dependent RNA polymerase), E (envelope) and N (nucleocapsid); the test was considered positive and the patient infected, if at least one gene was detected.

**Table 1:** Clinical indications/guidelines to perform SARS-CoV-2-RT-PCR on nasopharyngeal swabs.

Clinical indications/guidelines to perform SARS-CoV-2-RT-PCR on nasopharyngeal swabs
Fever (> 37.5^°^C) of unknown origin
Flu-like (rhinitis, conjunctivitis, cough) and/or pneumonia-like symptoms
Symptoms of gastrointestinal infection
Headache and/or anosmia and/or dysgeusia
Symptoms (also mild)+ history of close contacts with SARS-CoV-2 positive people
Impairment of general condition, hypotension, meningism, and/or signs of organ failure
Hospitalization for any reason (From April)

In this multicenter retrospective study we analyzed the medical charts of all included patients for demographic and clinical data, focusing on citizenship, anthropometry, co-morbidities, hospitalization length and short-term outcomes. As ethnicity is not recorded on hospital admission and citizenship in Italy is defined by *Jus sanguinis* – precluding children of foreign parents born in Italy the Italian citizenship until the age of 18 years - we used foreign citizenship as a proxy and defined ethnic groups according to geographical origin, using the ISTAT regions thus identifying European, African, Asian, Oceanian and American cohorts. We analyzed the impact of this so defined ethnicity on the frequency of SARS-CoV-2 infections by comparing the number of infected foreign children requiring hospital care with that of infected Italian children using the most recent total of foreign and Italian children living in the five provinces and calculated odds ratios. We then did the same comparisons among the different ethnic groups. We used the WHO Child Growth standards, especially male and female weight-for age Z-scores (SDS) (17) as uniform and reproducible parameter of patient's weight-associated risk factor (overweight and/or obesity) for COVID-19 incidence. Finally, we analyzed length of in-hospital stay of infected patients as a proxy-marker for severity of the infection.

### Statistical Analysis

Comparison for categorical variables (sex, ethnicity) was done using the chi-square test, differences in continuous variables with Gaussian distribution (age, Z-score for weight for age) were analyzed by the Student *t*-test, while for the comparison of variables with non-Gaussian distribution (length of stay) the Mann Whitney test was used. The level of significance for all statistical analysis was defined as an α of 0.05.

## Results

The total pediatric population in the five provinces as of December 2019 was 550,180 children under 16 years-old with 99,127 of them being foreigners. Among all children, we identified a total of 111 inpatients (51 females, 60 males) of whom 40 were hospitalized in Bergamo, 30 in Brescia, 15 in Pavia, 15 in Parma, and 11 in Cremona. Mean age was 5.2 years (range 1 day to 15 years), no significant difference between sexes.

**Figure 1:**
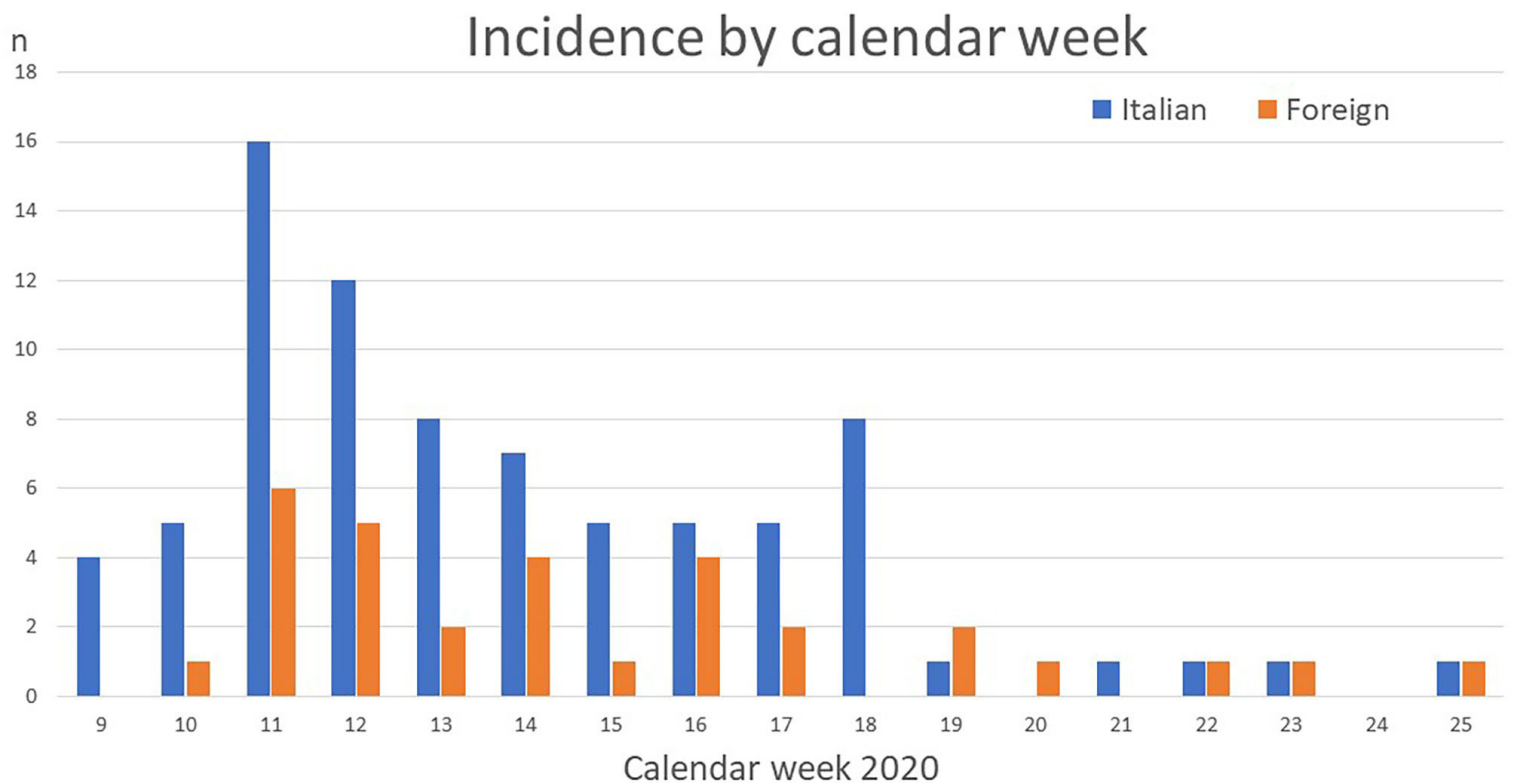
Shows the number of SARS-CoV-2 positive hospitalizations for Italian and foreign children over time.

There were 80 patients with Italian nationality and 31 of foreign nationality, among the latter there were 14 patients from Africa, 10 from Europe, four from Asia, and three from America. As from Oceania and among stateless children no positive patient was identified and being their share among all foreigners below 0.0% in all five provinces, we omitted this category from the presentation. Figure 1 shows the incidence of hospitalizations by ethnicity during the study period.

Distribution of the different ethnicities among Provinces and the respective hospitals is shown in Table 2, together with demographic and clinical characteristics.

**Table 2:** Shows the demographic distribution of the study population, clinical data of the hospitalized patients, and the results of the statistical analysis between all foreign patients combined vs. the Italian citizens (BG, Bergamo; BS, Brescia; CR, Cremona; PR, Parma; PV, Pavia; n.s., not statistically significant; SDS, standard deviation scores).

**Province**	**Africa**	**America**	**Asia**	**Europe**	**Foreigners combined**	**Italy**	***p***
	Total pediatric population of the catchment area (n)						
	28,552	6,370	20,724	43,494	**99,127**	**451,053**	
	SARS-CoV-2 positive hospitalized patients (n)						
BG	3	2	1	1	**7**	**33**	**n.s**
BS	2	1	1	2	**6**	**24**	**n.s**.
CR	2	0	2	0	**4**	**7**	**n.s**.
PR	5	0	0	3	**8**	**7**	**0.001**
PV	2	0	0	4	**6**	**9**	**0.031**
Combined	14	3	4	10	**31**	**80**	**0.006**
Female/male	7/7	1/2	2/2	4/6	**14/17**	**37/43**	**n.s**.
Mean/median age of patients (years)	4.2/3.1	3.4/0.76	6.7/6.4	6.6/6.4	**5.2/3.3**	**5.2/3.7**	**n.s**.
Mean SDS weight for age	+0.37	+0.5	−0.23	+0.81	**+0.45**	**+0.05**	**n.s**.
Mean/median length of stay (days)	10.3/7.5	8.6/8	15/16.5	8.9/5.5	**10.2/7**	**8.2/7**	**n.s**.
Comorbidities	7	8	0	1	**3**	**19**	**n.s**.

*Bold values are used for the combination of different ethnicities*.

**Figure 2:**
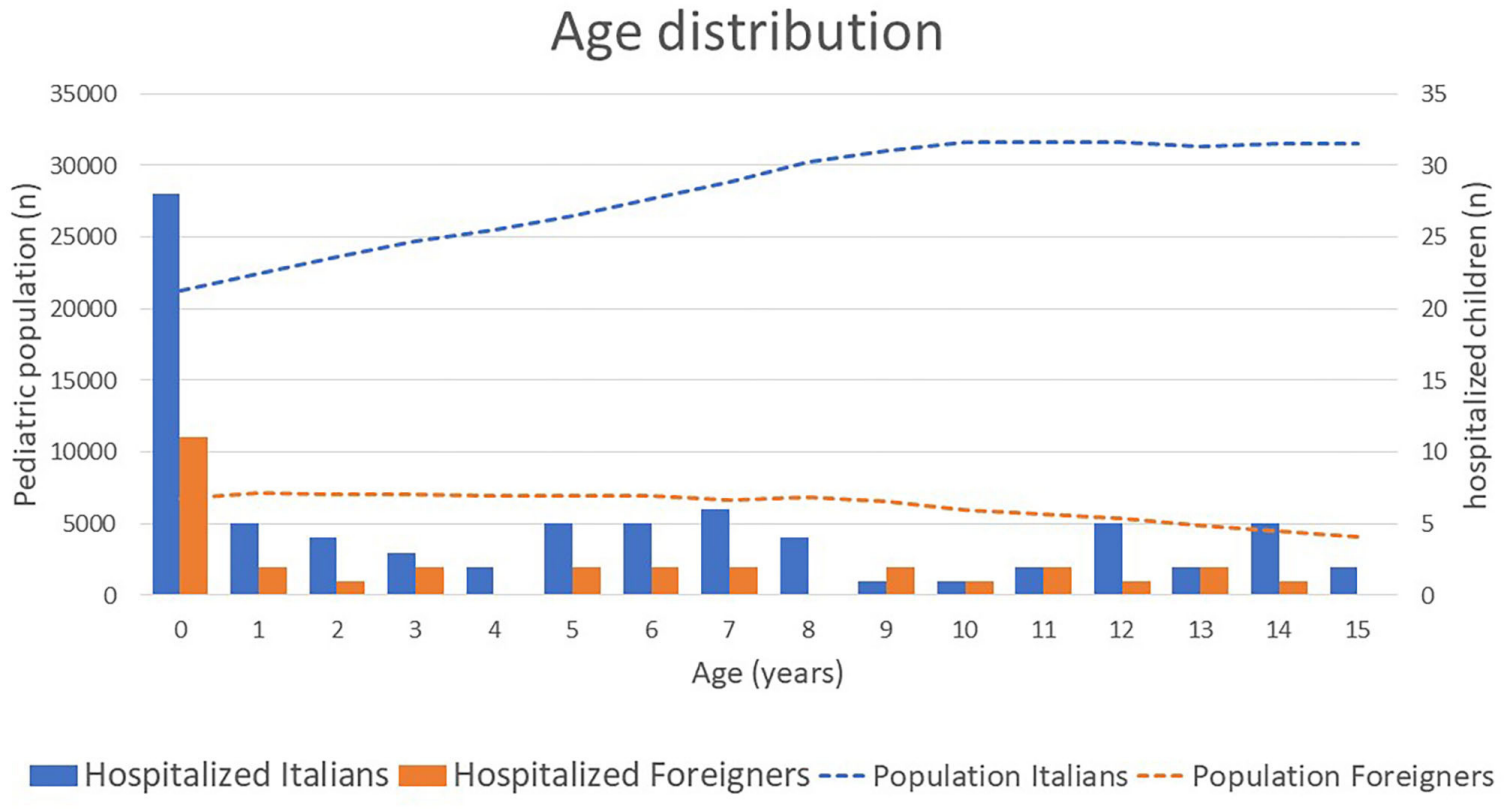
Shows the age distribution of the total pediatric population (lines) and the respective number of hospitalized SARS-CoV-2 positive children (bars).

Figure 2 shows the age distribution. Only in the Italian population there is an increment with increasing age reflecting a decrease in the birth rate over time which contrasts with the numbers seen in the foreign population. Among the hospitalized children of all ethnicities the youngest have a significantly increased risk for being hospitalized with SARS-CoV-2 infection (odds ratio for those <1 year against older children: 10.10, 95% CI: 6.84–14.92). However, there was no significant difference in age and sex distribution between foreign and Italian children, nor by comparing the different ethnicities.

The ratio of hospitalized foreign patients (31 out of a population of 99,127) was significantly (*p* = 0.006) higher than expected when compared with the Italian patients (80 out of 451,053) in all provinces (Table 2), leading to an odds ratio of 1.76 (95% CI: 1.16–2.66). Looking at the five provinces separately, this difference reached statistical significance also in Pavia (*p* = 0.03) and Parma (*p* = 0.001).

When analyzing the different ethnicities, we found patients from Africa to be the most affected: 0.49% of the pediatric African population were hospitalized with COVID-19, as compared to 0.47% of the American, 0.22% of foreigners from other European countries than Italy, 0.19% of the Asian and 0.17% of the Italian pediatric population. Only the difference between African and Italian children reached statistical significance (*p* = 0.0002), again with a significant odds ratio of 2.76 (95% CI: 1.56–4.87), while no difference was found between the other foreign ethnicities and the Italian patients. However, children from Africa had significant higher odds of being hospitalized with SARS-CoV-2 infection than all other foreign ethnicities combined (odds ratio 2.03, 95% CI: 1.003–4.13).

In order to understand if the higher hospitalization rate also corresponded to a more severe disease-course, we analyzed the length of hospitalization in different populations. The overall mean length of stay in the hospital was 8.8 days (median 7, interquartile range 8 days). Comparing length of hospitalization between foreign and Italian children we found the mean to be 2 days shorter (10.2 vs. 8.2 days) in the latter, but that difference was not statistically significant (median 7 days both groups, *p* = 0.6). A comparison limited to the African children in comparison to the Italian patients showed the same results. A separate analysis for the other ethnicities did not reveal significant differences. Also, there was no significant difference between the two sexes.

As obesity is a well-recognized risk-factor for COVID-19 (18, 19), we also calculated the Z-score for weight for age for all children and compared the value of Italian children (+0.05) with that of the other ethnicities (+0.45, *p* = 0.37). Again, the value for the African children (+0.37) did not differ significantly neither from the Italian patients (*p* = 0.31), nor from that of the other foreign children (+0.51, *p* = 0.69). There were more children with an SDS > 2 (7/80) among the Italian patients as compared to the foreign patients (2/31 – both European), but the difference was not significant (*p* = 0.69). As infants were more frequently hospitalized than older children, we compared body weight among the two groups, but did not find any difference in standard deviation scores (*p* = 0.33).

The average incidence of comorbidity, another confounder for hospitalization in SARS-CoV-2 infected children revealed no major differences between the different ethnic groups: 16.25% (13 of 80) of Italian patients had comorbidity as compared to 19.35% (6 of 31) of foreign patients (*p* = 0.74). Among the latter the percentages were 30% (3 out of 10) for European and 21.2% (3 out of 14) for African children, while among the 4 Asians and 3 American children no comorbidity occurred. None of those differences were statistically significant.

Thus, the rate of African children hospitalized for SARS-CoV-2 infection during the first wave of the COVID-19 pandemic in Northern Italy was higher than that of any other ethnic group. However, an increased Z-score for bodyweight does not seem to be responsible for the difference observed and apparently there is no difference in disease severity as measured by length of hospitalization.

## Discussion

Eight months after the outbreak of the COVID-19 pandemic, data on SARS-CoV-2 infection in children of different ethnicities and different ages are still lacking (20). In our study, to our knowledge the most numerous to provide ethnic, demographic, and epidemiological data from a purely pediatric population hospitalized for COVID-19 during the first wave of the pandemic, we analyze data from five provincial HUB Centers in the heart of the first epidemic wave in Northern Italy.

In comparison to adults, children seem to have an asymptomatic or at least milder course of the infection in most cases (21, 22), although age <1 year – as confirmed by our own data - and the presence of underlying medical conditions have been associated with more severe manifestations, requiring intensive care and sometimes fatal outcome (11, 23, 24). In the largest study published on COVID-19 in children in Italy (25), of 3,836 infected children only 13.3% needed hospitalization, a percentage which applied to our study, would bring the total of infected children in our catchment area to 723, or 0.7% of the total pediatric population – a scenario in line with the positive rate of 0.9% of the total Lombard population (94,905 out of 10,103,000) found infected by July 10.

We describe a higher rate of male patients in this age group as compared to infected adults in Italy (14) but a slight majority of male children has been described in many national and international pediatric studies (18, 19, 21–23, 25–27).

Median age of our patients is 5.2 years and thus at the lower end of the range of most studies [5.0 (23) 7 years (21), 9 years (18), 11 years (25)]. However, most considered children up to the age of 18 or even 21 years - an age at which most patients in our area are hospitalized in adult wards, which explains the difference.

While in adults several reports from the USA and UK highlight the higher risk for infections with SARS-CoV-2 and more severe manifestations of COVID-19 including death, among black people and ethnic minorities (28), only few publications so far consider this aspect in the pediatric population. Furthermore, most of these report on only a few infected children, do not always differentiate between African, Latin or Asian populations (13). Almost all also include adolescents and young adults (18) and – in contrast to the data presented here - do not provide ethnic data of the general pediatric population, necessary for an analysis of the impact ethnicity has. Nevertheless, the percentages provided, ranging from 56% [14 black out of 25 in Chicago (10)] to 75% [9 BAME out of 12 in London (13)] and even 80% among those needing intensive care, underline the need for a broader dataset to be analyzed.

In our setting, we found a significantly higher prevalence of SARS-CoV-2 positive foreign children hospitalized than the one expected by comparing their prevalence with that of Italian children, using the official ISTAT data for the foreign and Italian pediatric population. The odds ratio for being hospitalized with a SARS-CoV-2 infection is highest for the African population: 2.76 (95% CI: 1.56–4.87), significantly higher as for the other foreign ethnicities, which in turn where not significantly more affected than the Italian population.

Apparently foreign children did not have a more severe disease-course, as there was no significant difference, once hospitalized, in the length of stay between Italian and foreign patients. As fortunately none of our patients died, we decided to use length of hospitalization as a proxy for disease severity in order to eliminate the bias of subjective clinic-based definitions (21). However, the need of hospitalization itself is probably a marker of disease severity, so that a certain bias cannot be completely excluded. There are however recent data from the UK (27) showing an increased risk for the pediatric inflammatory multisystem syndrome temporally associated with SARS-CoV-2 (PIMS-TS) in patients from ethnic minority backgrounds. However, detailed and comparable population data are missing in this report.

Among adults being overweight/obese is a major risk factor for severe disease, and especially for the need for mechanical ventilation. Being more frequent in the African American population, this risk factor has also been made partially responsible for the ethnic differences. In children the small study from London (13) showed obesity as a frequent comorbidity and a larger study on 50 children and young adults confirmed obesity to be a risk factor, especially in patients older than 2 years. But as this cohort included young adults up to the age of 21 years, the significance of this risk factor for children <16 years was not assessed (18).

We thus looked at the weight-for-age Z-score of our patients but did not find any significant difference between foreigners and Italians, nor between the different ethnicities. However, we cannot exclude that using the BMI instead of the weight-for-age SDS as an obesity indicator in older children could have given slightly different results, but the latter was not available for analysis.

Comorbidities (excluding obesity) were somewhat lower (17.1%) than what would be expected from the literature, where the rate among the hospitalized usually is between 20% (26) and over 60% (18). Higher rates are seen in patients needing Intensive care, again a condition not observed in our cohort. Neurologic and oncologic/immunodeficient conditions were the most frequent, which is in line with the expected. Other authors investigated asthma as a risk factor, but like us were unable to confirm this hypothesis (10). There was a slight difference in the frequency of comorbidities between African children (21.4%) as compared to the Italian children (16.3%). However, in contrast to published data from the USA (11) that difference was not statistically significant nor was there any significant difference between the ethnicities. The generally lower presence of comorbidities among our patients may reflect the fact that our data comprise the beginning of the pandemic in Italy, when there were probably more mild courses hospitalized than during the second phase, when only more severely ill children were hospitalized. This regards in particular patients with oncologic diseases, who were hospitalized because of fear (of parents and healthcare workers) that the – by then largely unknown course of the infection in immunocompromised children, might be much more severe than in the general pediatric population. Some of those patients also had a rather long in-hospital stay, but mostly due to chemotherapy courses that were administered during the same hospitalization.

In summary, besides age <1 year, we must confirm an increased risk for foreign, especially African children to be hospitalized with SARS-CoV-2 infection also in our setting.

Many suggestions have been made to explain those differences also from a genetic or physiologic point of view (29). However, it's surprising to note that Africa is in fact the least affected among the regions of the World Health Organization, thus pointing to other, possibly environmental factors. Surely many drivers of the pandemic are less important in the African continent, namely obesity and additional risky comorbidities, population density, crowded housing and last but not least the climate may contribute to explain (part of) the difference (9). Another explanation could be the presence of cross-reacting antibodies from previous (tropical?) infections that are differently prevalent on various continents and may play a role that could be protective (in Africa) or even cause antibody-dependent enhancement (in Europe). Recently, auto-antibodies against IFN have been found in severe cases of COVID-19, which also may have an ethnicity-linked prevalence (30). Finally, a further hypothesis, quite relevant to African children in Europe considers low Vitamin D levels to be a risk factor, especially for PIMS-TS (31). Thou current consensus from the Italian Society for Preventive Medicine and other bodies recommend supplementation with Vit D for children and adolescents with dark skin living in Europe (32), data on the uptake of this recommendation are missing.

Unequal access to healthcare has been linked to distance from a hospital, family income and insurance status, problems well-known in the USA, but probably of lower impact in Italy, where the constitution in theory and the Italian National Health Service in practice guarantees equal healthcare levels free of charge to all children regardless of their ethnicity, their social or economic situation.

However, socio-economic status is a factor to consider also in Italy. The last ISTAT reports on national poverty and education show that the 6.3% of Italian families and the 31.2% of foreign families in Italy are below the threshold of absolute poverty (33). This may influence the living conditions in general (space, heating, food), the possibility to stay at home with a sick child or to pay for transport. Cultural barriers and health-literacy are other important factors to consider: 64% of Italian adults have at least a High School diploma, as compared to only 47.3% of foreign adults in Italy (34). These data are significant in an epidemic, where proper understanding of how to prevent contagion is essential – and existing language barriers may be enhanced by face masks.

The study has several weaknesses: since ethnicity and citizenship are not the same, we cannot completely exclude the presence of children of non-Caucasian origin among the Italians and vice-versa. However, as in Italy citizenship is still acquired through *Jus sanguinis* and recorded as such by the national bureau of statistics, the number of erroneously attributed ethnicity is probably small and evenly distributed among patients and the general population. Another limit is that the admission and even more the discharge criteria may have varied during the first wave, as beds started to become scarce and situations at home (other infected, hospitalized, or quarantined family members) had to be taken into account.

The strength of this study is the huge clearly defined pediatric population and the strict age-limit, which allows meaningful conclusions for pediatricians.

In conclusion, our study is the first large population-based report to demonstrate an effect of ethnicity on SARS-CoV-2 infection-related hospitalizations in children at the heart of this ongoing pandemic. More research is needed to better understand and quantify the underlying reasons and find ways to overcome them in near future!

## Data Availability Statement

The raw data supporting the conclusions of this article will be made available by the authors, without undue reservation.

## Ethics Statement

Ethical review and approval was not required for the study on human participants in accordance with the local legislation and institutional requirements. Written informed consent from the participants' legal guardian/next of kin was not required to participate in this study in accordance with the national legislation and the institutional requirements.

## Author Contributions

RFS had the idea, supervised data collection, made statistical analysis, and wrote the final manuscript. RB, LS, JR, and BC collected data, drafted the manuscript, and prepared tables and figures. AM, GM, ID, and CC provided data. All authors participated in the writing of the manuscript and approved the final version.

## Conflict of Interest

The authors declare that the research was conducted in the absence of any commercial or financial relationships that could be construed as a potential conflict of interest.
